# Excess membrane binding of monomeric alpha-, beta- and gamma-synuclein is invariably associated with inclusion formation and toxicity

**DOI:** 10.1093/hmg/ddab188

**Published:** 2021-07-12

**Authors:** Tae-Eun Kim, Andrew J Newman, Thibaut Imberdis, Lisa Brontesi, Arati Tripathi, Nagendran Ramalingam, Saranna Fanning, Dennis Selkoe, Ulf Dettmer

**Affiliations:** Ann Romney Center for Neurologic Diseases, Department of Neurology, Brigham and Women’s Hospital and Harvard Medical School, Boston, MA 02115, USA; Ann Romney Center for Neurologic Diseases, Department of Neurology, Brigham and Women’s Hospital and Harvard Medical School, Boston, MA 02115, USA; Ann Romney Center for Neurologic Diseases, Department of Neurology, Brigham and Women’s Hospital and Harvard Medical School, Boston, MA 02115, USA; Ann Romney Center for Neurologic Diseases, Department of Neurology, Brigham and Women’s Hospital and Harvard Medical School, Boston, MA 02115, USA; Ann Romney Center for Neurologic Diseases, Department of Neurology, Brigham and Women’s Hospital and Harvard Medical School, Boston, MA 02115, USA; Ann Romney Center for Neurologic Diseases, Department of Neurology, Brigham and Women’s Hospital and Harvard Medical School, Boston, MA 02115, USA; Ann Romney Center for Neurologic Diseases, Department of Neurology, Brigham and Women’s Hospital and Harvard Medical School, Boston, MA 02115, USA; Ann Romney Center for Neurologic Diseases, Department of Neurology, Brigham and Women’s Hospital and Harvard Medical School, Boston, MA 02115, USA; Ann Romney Center for Neurologic Diseases, Department of Neurology, Brigham and Women’s Hospital and Harvard Medical School, Boston, MA 02115, USA

## Abstract

α-Synuclein (αS) has been well-documented to play a role in human synucleinopathies such as Parkinson’s disease (PD) and dementia with Lewy bodies (DLB). First, the lesions found in PD/DLB brains—Lewy bodies and Lewy neurites—are rich in aggregated αS. Second, genetic evidence links missense mutations and increased αS expression to familial forms of PD/DLB. Third, toxicity and cellular stress can be caused by αS under certain experimental conditions. In contrast, the homologs β-synuclein (βS) and γ-synuclein (γS) are not typically found in Lewy bodies/neurites, have not been clearly linked to brain diseases and have been largely non-toxic in experimental settings. In αS, the so-called non-amyloid-β component of plaques (NAC) domain, constituting amino acids 61–95, has been identified to be critical for aggregation *in vitro*. This domain is partially absent in βS and only incompletely conserved in γS, which could explain why both homologs do not cause disease. However, αS *in vitro* aggregation and cellular toxicity have not been firmly linked experimentally, and it has been proposed that excess αS membrane binding is sufficient to induce neurotoxicity. Indeed, recent characterizations of Lewy bodies have highlighted the accumulation of lipids and membranous organelles, raising the possibility that βS and γS could also become neurotoxic if they were more prone to membrane/lipid binding. Here, we increased βS and γS membrane affinity by strategic point mutations and demonstrate that these proteins behave like membrane-associated monomers, are cytotoxic and form round cytoplasmic inclusions that can be prevented by inhibiting stearoyl-CoA desaturase.

## Introduction

Ever since α-synuclein (αS) was identified to be a primary component of Lewy bodies and Lewy neurites (1) in synucleinopathies, such as Parkinson’s disease (PD), dementia with Lewy Bodies (DLB) and multiple-system atrophy (MSA), the endeavors of many laboratories have coalesced around elucidating αS native structure, physiological function, aggregation propensity and cell toxicity. Our understanding of the remaining two homologous proteins in the synuclein family, β-synuclein (βS) and γ-synuclein (γS), however, remains more nebulous than our knowledge of αS. No reports have directly implicated βS or γS mutations and associated aggregation to a synucleinopathy and neither protein is typically considered to be a major presence in Lewy bodies or Lewy neurites (2–4). However, occasional reports have identified βS and/or γS in synucleinopathy lesions, albeit not classical Lewy bodies (5–7). Moreover, a study that screened and sequenced DLB patient and family samples identified two mutations to βS, V70M and P123H, to be potentially associated with αS aggregation. The authors speculated that either mutant might interfere with an unknown molecular pathway in which normal βS prevents αS aggregation (8). γS finds itself most distant from synucleinopathy research because, unlike αS and βS (9), it is prevalent in the peripheral nervous system (10,11). In addition, γS has been identified in liver, breast carcinoma and possibly ovarian tumors (11,12).

**Figure 1:**
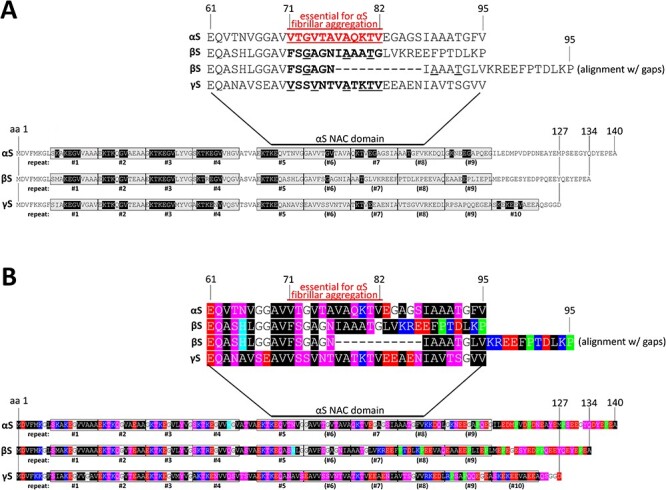
(**A**) Schematic of aligned αS, βS and γS amino acid sequences. Bottom, amino acids were aligned from N-terminus to C-terminus and demarcated with the up to 10 repeat motifs. Amino acids highlighted in black are conserved with regard to the KTKEGV core repeat motif. Top, close-up of the region that is called ‘NAC domain’ for αS, with a special emphasis of amino acids 71–82 that had been reported to be essential for αS aggregation. βS is characterized by a deletion relative to αS and γS in this region, and we are displaying both an alignment with and without a gap. (**B**) Schematic of aligned αS, βS and γS aligned by repeat motif, color-coded. Analogous to (A) but color-coded: blue indicates basic (light blue: histidine); red: acidic, purple: polar uncharged and black: non-polar, green: proline residues.

Despite being localized in various cell types and involved in various diseases, all three synucleins have a considerable degree of sequence and structural similarities (Fig. 1A and B). They generally share a similar N-terminal region of which αS has been purported to form a single or two distinct α-helices when interacting with the lipid bilayer of membranes (13–17) and a C-terminal region that is populated with negatively charged residues and prolines (Fig. 1A and B). Amino acid positions 61–95 were identified in αS as the ‘non-amyloid-β component’ (NAC) of Alzheimer’s disease amyloid plaques (18,19). In particular, amino acids 71–82 within the αS NAC region have been proposed to be critical for β-sheet-rich aggregation *in vitro* (20,21). This region consists of VTGVTAVAQKTV in αS, FSGAGNIAAATG in βS and VSSVNTVATKTV in γS (Fig. 1A; conserved amino acid relative to αS are underlined). Giasson *et al*. (21) postulated that the 71–82 stretch is both necessary and sufficient for αS fibrillization based on the following observations: (1) amino acids 71–82 are not conserved in βS, and βS does not assemble into filaments *in vitro*; (2) introducing a single charged amino acid within these 12 residues reduces αS *in vitro* aggregation, and a deletion of the 12 amino acid abrogates it; (3) the stretch resists proteolytic digestion in αS filaments and (4) synthetic VTGVTAVAQKTV peptides form filaments *in vitro* and promote fibrillization of full-length αS.

**Figure 2:**
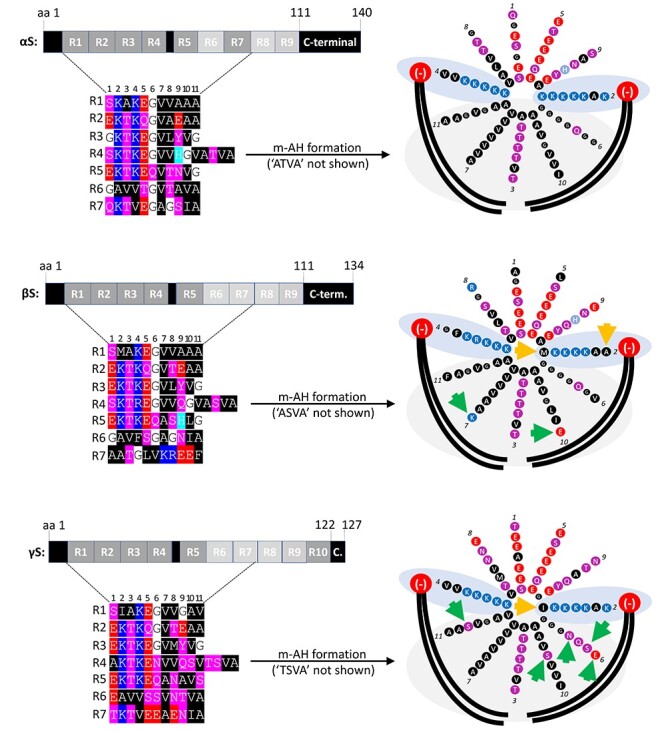
Left, schematic of αS, βS and γS aligned by the first seven repeat motifs. αS, βS and γS are depicted as a series of seven repeats R1–R7, and amino acids are color-coded. Blue indicates basic (light blue: histidine); red: acidic, purple: polar uncharged and black: non-polar residues. Right, αS membrane-induced amphipathic helix (m-AH) formation (αS, βS and γS helical wheels embedded in membrane lipids). The formation of 3–11 helices (3 turns per 11 amino acids) at membranes is driven by hydrophobic non-polar amino acids in αS that interact with fatty acid tails of membrane lipids (large black curved lines) and by positively charged lysine (K) residues that interact with negatively charged lipid head groups of membrane lipids (large red circles). Arrows indicate amino acid substitutions relative to αS that are expected to lower membrane binding by either reducing electrostatic (yellow) or hydrophobic interactions (green).

While key elements of the NAC domain—including the 71–82 amino acid region—seem to be homolog-specific, a defining feature of all synucleins is a 11-amino acid repeat with the core consensus motif KTKEGV. The 11-amino acid repeat spans several times in αS, βS and γS throughout the N-terminus, albeit with slight differences (Fig. 1A and B). In αS, repeats #1–5 and 7 are highly conserved, in βS, repeats #1–5 are highly conserved. γS seems to contain conserved repeats #1–5 and 7 plus an additional relatively conserved KTKEGV motif near its C-terminus that is not mirrored in αS or βS (Fig. 1A and B). There have been several studies relating αS aggregation with cell toxicity and pathology from the perspective of the repeat motif (22–26) as the motif seems to be critical for αS’s normal dynamic behavior in cells (reviewed in 27). Our group has contributed to an increasingly nuanced understanding of αS dynamics in which the KTKEGV motif plays a central role (28–30). We proposed that: (1) αS physiologically exists in dynamic and sensitive equilibria between cytoplasmic versus membrane-associated species as well as monomeric versus multimeric species; (2) disruption of these dynamic αS equilibria results in cell toxicity; (3) specific missense mutations to the αS KTKEGV repeat motif can alter the equilibria to the cell’s detriment by stabilizing αS amphipathic helices at membranes and rendering αS unable to multimerize and (4) cytotoxic αS KTKEGV variants cause vesicle- and lipid-droplet-rich inclusions largely devoid of apparent fibrillar material, as assessed by electron microscopy (EM), while crosslinking and yellow fluorescent protein (YFP) complementation analyses were consistent with monomeric—not aggregated—αS being present in the inclusions (30–32). The hypothesis that we have maintained confers the KTKEGV repeat motif as an important component in αS membrane binding, multimerization and toxicity.

Here, we report that rationally designed KTKEGV repeat motif mutants in αS, βS and γS can invariably be associated with inclusion formation and toxicity in neural cells. The biochemical characterization of the engineered mutants suggests the accumulation of monomeric proteins at membranes. Our observations are consistent with a membrane-mediated mechanism of synuclein toxicity that is independent of the αS NAC domain and may also (at least initially) be independent of proteinaceous, β-sheet-rich aggregation.

## Results

### Design of αS, βS and γS variants with strong effects on membrane binding and multimerization

**Figure 3:**
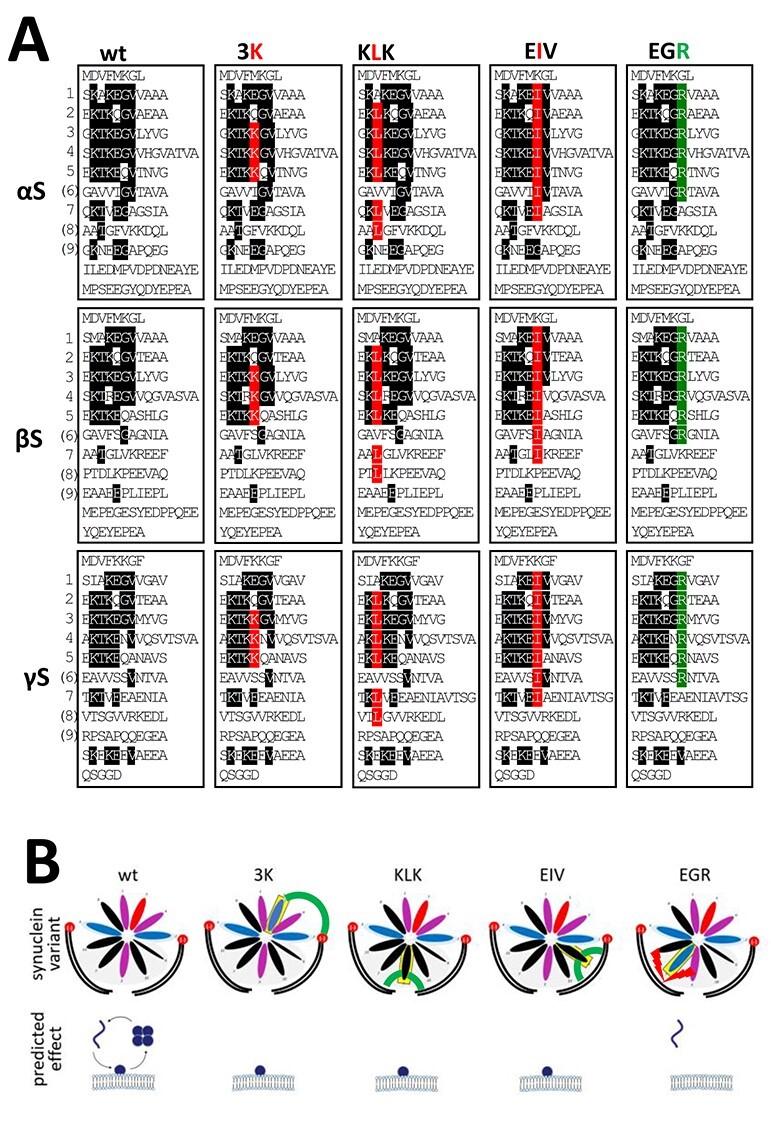
(**A**) Schematic of wt and strategic missense mutations of αS, βS and γS. Sequences are aligned by their KTKEGV repeat motifs. Amino acids that fully conform to the KTKEGV repeat are highlighted in black. Amino acids in red are the strategic missense mutations that disrupt equilibria toward membrane-bound monomers. Amino acids in green are missense mutations that are expected to disrupt equilibria toward cytosolic monomers. (**B**) Schematic of predicted changes in equilibria. Among cytosolic monomer, cytosolic multimer and membrane-bound monomer for all synuclein species, 3K, KLK and EIV are expected to strongly reduce cytosolic species levels (K interacts with phospholipid head, L and I interact with lipid tails), whereas EGR is predicted to remain largely cytosolic (R repels αS from membrane lipid tails).

We have recently shown that strategic missense mutations to the repeat motif of αS [exaggerations of familial PD (fPD)-linked mutants or strategic designs] can alter the protein’s membrane interactions and assembly state. Four such αS mutant variants that we extended to βS and γS are relevant for this study (Fig. 3A): (1) three KTKEGV→KTKKGV mutations which we named ‘3K’. In the case of αS, this variant is an amplification of the αS fPD E46K mutant (E35K + E46K + E61K). 3K presumably stabilizes membrane-induced synuclein helices by providing additional attraction between positively charged lysine residues and negatively charged lipid headgroups (33,34); see Fig. 2. We had previously found decreased αS cytosol:membrane ratios for αS 3K, which were accompanied by decreased multimer:monomer ratios, increased inclusion formation and increased toxicity (31). We now generated βS 3K and γS 3K (both are E35K + E46K + E61K) for comparison (Fig. 3A, second column). (2) Six KTKEGV→KLKEGV mutations that we named ‘KLK’ (30). KLK presumably strengthens hydrophobic interactions with membrane lipids by replacing threonine with leucine residues, thereby increasing the hydrophobicity of the membrane-embedded half of the synuclein amphipathic helix and giving rise to additional steric interactions. We had previously found strongly decreased αS cytosol:membrane ratios for αS KLK that were accompanied by decreased multimer:monomer ratios, increased inclusion formation and increased toxicity (30). We now generated βS KLK and γS KLK for comparison (Fig. 3A, third column). (3) Seven KTKEGV→KTKEIV mutations which we named ‘EIV’ (30). Like KLK, this variant increases the hydrophobicity of the membrane helix (Fig. 2A, fourth column) and (4) six KTKEGR mutations which we named ‘EGR’ (30). EGR has additional positive arginine charges in the hydrophobic half of the helix (Fig. 3B, last column, top). Based on previous work on similar helix-disrupting αS mutations (35,36) and a reported mechanism of αS multimer formation that involves transient membrane interactions (37), it is plausible that αS EGR is repelled from membranes and accumulates in the cytosol as an (unfolded) monomer (Fig. 3B, last column, bottom). Here, we generated βS EGR and γS EGR for comparison with αS EGR.

Given similar sequences across all synuclein species, and given our previous report demonstrating that wt αS, βS and γS (Fig. 3A, first column) all exhibit a pattern of apparent multimers and monomers upon crosslinking (30), it seemed reasonable to hypothesize that the phenotypic differences among the various missense mutations introduced in αS might also hold true if we introduced the analogous mutations in βS and γS (Fig. 3A).

### The conserved KTKEGV repeat motifs mediate αS, βS and γS membrane interaction and assembly

**Figure 4:**
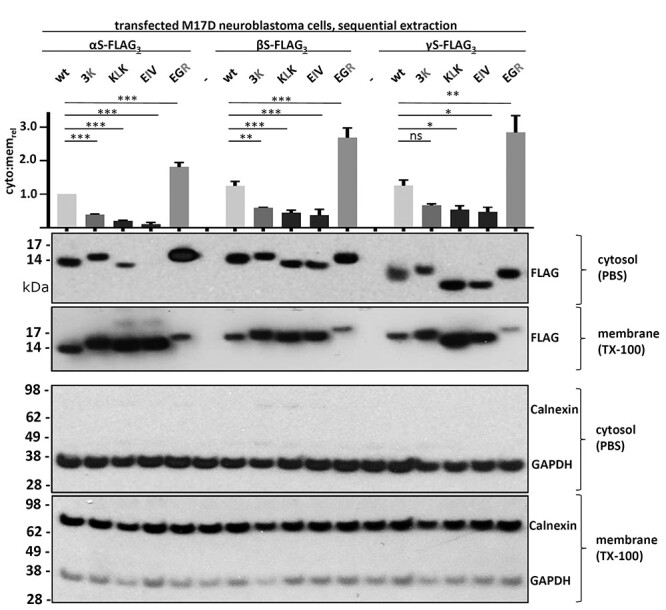
WB of sequential protein extraction and quantification. All human synuclein homologs, wt and the indicated variants were transiently transfected in M17D neuroblastoma cells. Cytosolic (PBS-soluble) and membrane proteins (TX-100-soluble) were separated by sequential extraction. Synucleins were detected using anti-FLAG (first and second WB rows, respectively). Controls for cytosolic and membrane fractions were GAPDH and Calnexin, respectively. WB is representative of *N* = 3 independent transfections on different days. Cytosol:membrane ratios were quantitated relative to αS wt, which was set to 1. We determined for each variant if cytosol:membrane ratios were significantly different versus the respective wt. Graph shows mean data for *N* = 3 independent experiments and standard errror of means (SEM). One-way analysis of variance (ANOVA) analysis, Tukey’s multiple comparisons test. ^*^P < 0.05; ^*^^*^P < 0.01; ^*^^*^^*^P < 0.001; ns, non-significant.

**Figure 5:**
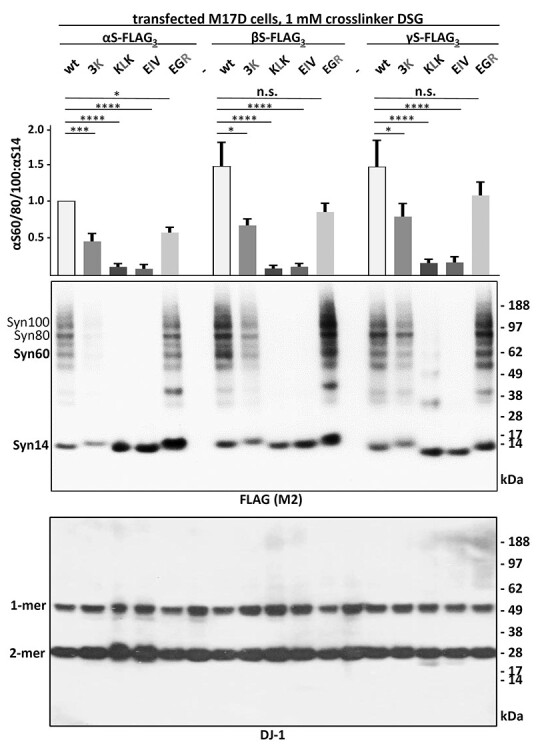
WB and quantitation of αS60/80/100:αS14 ratios. Synuclein homologs and variants were transfected in M17D cells. Anti-FLAG WB after intact cell crosslinking (top). Blotting for DJ-1 served as a control (bottom). The ratios of Syn60/80/100 and Syn14 were normalized to αS wt and quantitated relative to each respective wt. Graph shows mean data for *N* = 3 independent experiments performed on different days in duplicates (*n* = 6) and SEM. One-way ANOVA analysis, Tukey’s multiple comparisons test. ^*^P < 0.05; ^*^^*^^*^P < 0.01; ^*^^*^^*^^*^P < 0.001; n.s., non-significant.

To test whether the previously described effects of αS 3K, KLK, EIV and EGR mutagenesis might apply to all synuclein homologs, we expressed the respective αS, βS and γS variants in M17D neuroblastoma cells by lipofection. We previously demonstrated the multimerization of endogenous neuronal αS and βS by intact-cell crosslinking (30). Moreover, we showed by sequential extraction that endogenous neuronal βS is less membrane-associated than αS, while both homologs are present in cytosol and membrane fractions in relevant amounts (38). To enable direct comparisons across all three synuclein proteins and their variants in the present study, we fused our constructs with C-terminal FLAG_3_ tags. 48 hours post-transfection, we subjected the cells to sequential protein extraction and analyzed PBS-soluble (‘cytosol’) and detergent-soluble (1% TX-100; ‘membrane’) fractions by Western blot (WB). As expected, based on our previous work, we found αS 3K, KLK and EIV to be enriched in the membrane fraction, whereas EGR accumulated in the cytosol (Fig. 4). Overall, we observed similar effects in the respective βS and γS mutations, with only γS 3K not being significantly different from wt under the chosen conditions (Fig. 4). 3K had weaker effects than KLK or EIV with regard to increasing αS membrane binding, which might be explained by only three residues being changed in comparison to six and seven in the cases of KLK and EIV, respectively. Next, we asked whether introducing the 3K, KLK, EIV or EGR mutations into βS and γS would also affect multimer:monomer ratios, as we had observed for αS (30,31,39). As in our previous studies, we tested for synuclein assembly by intact-cell crosslinking using 1 mM disuccinimidyl glutarate (DSG) and WB (Fig. 5). We observed that: (1) all wt and EGR variants exhibited both multimers [30, 60, 80 and 100 kilodalton (kDa)] and monomers (14 kDa), but the multimer:monomer ratios of the EGR variants were reduced relative to wt; (2) all 3K variants had lower levels of multimers when compared with their wt counterparts and (3) KLK and EIV variants were predominantly monomeric as expected from our previous αS studies (30,39). To confirm one of our key observations in the absence of protein tags, we subjected untagged αS and βS wt and 3K variants to DSG crosslinking followed by sequential extraction. In both homologs, the 3K variants were more membrane-associated and exhibited reduced 60/80/100 kDa multimers, which were mostly found in the cytosolic fractions for both wt and 3K. Conversely, monomer accumulation was observed in membrane fractions (Supplementary Material, Fig. S1).

### Decreased solubility and multimerization in αS, βS and γS are associated with toxicity

**Figure 6:**
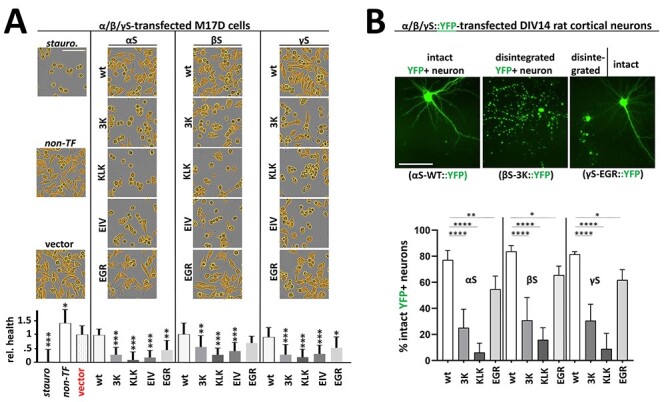
(**A**) Live-cell imaging of M17D cell confluence. M17D cells transfected with FLAG_3_-tagged wt, 3K, KLK, EIV or EGR variants for all human synuclein homologs. Staurosporine-treated (a strong toxin, positive control, set to 0 viability), non-transfected and vector-transfected cultures (both negative controls) are shown in the left column. Images were taken 48 h post-transfection by IncuCyte live-cell imaging. Cells were identified and their confluence quantified by using a custom IncuCyte algorithm that identifies areas occupied by cells (displayed in orange). Images are representative of *N* = 4 independent experiments performed in quadruplicates (*n* = 16). Graph shows mean data and standard deviation. All statistics relative to vector only (red). One-way ANOVA analysis, Tukey’s multiple comparisons test. ^*^P < 0.05; ^*^^*^P < 0.01; ^*^^*^^*^P < 0.001. Scale bar, 50 µm. (**B**) Assessing neuron integrity upon transfection of YFP-tagged synuclein variants. YFP-tagged wt, 3K, KLK or EGR variants for all human synuclein homologs were transfected into DIV14 rat cortical neurons. Transfection efficiency <5% allowed for assessing integrity of single transfected neurons 96 h post-transfection. After image acquisition and blinding, cells were categorized into ‘intact’ and ‘disintegrated’ and the relative percentage of intact neurons was calculated (*N* = 3 independent experiments, *n* = 2 transfected wells per experiment and variant, 36 fields per well). Graph shows mean data and standard deviation. All statistics relative to the respective wt variant. One-way ANOVA analyses, Tukey’s multiple comparisons test. ^*^P < 0.05; ^*^^*^P < 0.01; ^*^^*^^*^^*^P < 0.0001. Scale bar, 50 µm.

We previously reported that inclusion formation owing to certain engineered αS KTKEGV repeat motif mutations is associated with cell stress/toxicity (30,31,39,40), and we planned to verify if this also held true when analogous mutations were introduced into the repeat motifs in βS and γS. We therefore transfected M17D cells with all of our FLAG_3_-tagged variants and observed by live-cell imaging how overexpression of the various synuclein variants might affect cell confluence as a proxy for cell health. 48 hours post-transfection, the confluence of M17D cells that were transfected with wt synucleins was not significantly different from those that were transfected with vector alone. However, the relative confluence of all 3K, KLK and EIV transfectants was significantly lower (Fig. 6A). The reduced confluence was accompanied by changes in cell morphology: many cells appeared rounded and detached, similar to cells that were treated with the strong toxin staurosporine (Fig. 6A, top-left image). This indicated to us that 3K, KLK and EIV variants were toxic for all synuclein homologs. The EGR variant was generally less toxic than the other variants (as indicated by higher confluence levels and fewer numbers of small and rounded cells); its confluence level was moderately less than wt (Fig. 6A). We next transfected days *in vitro* (DIV) 14 rat neurons with the respective YFP-tagged proteins (wt, 3K, KLK and EGR) and recorded YFP signals in the IncuCyte live-cell microscope after 96 h. Owing to the relatively low transfection efficiency, we were able to identify single YFP+ neurons and categorized them into intact and disintegrated neurons (see Fig. 6B, top image for examples). Blinded analysis revealed that for all synuclein homologs, about 80% of wt YFP+ neurons were intact, a value that was strongly reduced in the case of 3K and KLK (*P* < 0.0001) and slightly reduced in the case of EGR variants (*P* < 0.05). In the case of 3K and KLK, even seemingly intact neurons displayed changes in cellular αS distribution (inclusion formation), which we addressed in detail for shorter transfection times (see next paragraph).

### Decreased multimerization and solubility in αS, βS and γS are associated with cytoplasmic inclusion formation

**Figure 7:**
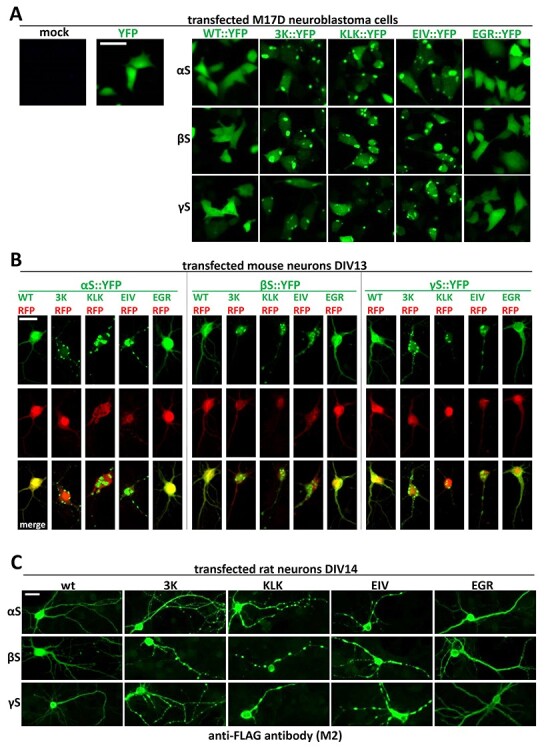
Fluorescence microscopy images of cells transfected with wt and variants of synuclein homologs. (**A**) M17D cells were transfected with YFP-tagged wt, 3K and KLK variants for all homologs. YFP was also transfected as a control. Images were taken 48 h post-transfection. Scale bar, 25 µm. (**B**) DIV 13 primary mouse neurons were co-transfected with YFP-tagged variants for all homologs and RFP as a control. Images were taken 48 h post-transfection. Scale bar, 20 µm. (**C**) DIV 14 rat neurons were transfected with YFP-tagged variants for all homologs. Images were taken 48 h post-transfection. All images are representative of *N* = 3 independent experiments done on different days. Scale bar, 20 µm.

Previously, we showed that transfecting M17D cells with αS 3K, KLK and EIV variants resulted in inclusion formation, whereas αS EGR, like wt αS, remained diffuse throughout the cytosol. We had also observed that primary rat neurons transiently transfected with αS 3K, KLK and EIV variants displayed multiple inclusions of various sizes in both somata and neurites (30,31); EM identified the inclusions as rich in vesicles and lipid droplets (32,39). Here, we first transiently transfected M17D cells with YFP-tagged wt and variant αS, βS and γS. We observed by IncuCyte live-cell imaging that all wt synuclein homologs remained diffuse throughout the cytosol and that all 3K, KLK and EIV variants formed round inclusions of various sizes 48 h post-transfection (Fig. 7A). We verified our observations by co-transfecting DIV 13 mouse neurons with YFP-tagged synuclein variants and red fluorescent protein (RFP) as a cytosolic marker and control for cell integrity: using fluorescence microscopy of fixed cells, we observed that, after 48 h, all wt and EGR constructs remained diffuse and cytosolic like RFP, whereas 3K, KLK and EIV expressions resulted in the appearance of multiple round inclusions in somata and neurites (Fig. 7B). Of note, the 3K, KLK and EIV inclusions (green signal) did not seem to co-localize with RFP (red signal). This is unlike the wt and EGR synuclein homologs, which overlapped with RFP (merged signals). Transient transfections of DIV 14 primary rat neurons with FLAG_3_-tagged wt and variants of all three homologs followed by immunofluorescence led to similar results (Fig. 7C), thereby confirming our previous observations: wt and EGR of all synuclein homologs appeared diffuse throughout the neuron, whereas 3K, KLK and EIV variants formed round inclusions.

### Pharmacological alteration of (membrane) lipid composition rescues synuclein inclusions

**Figure 8:**
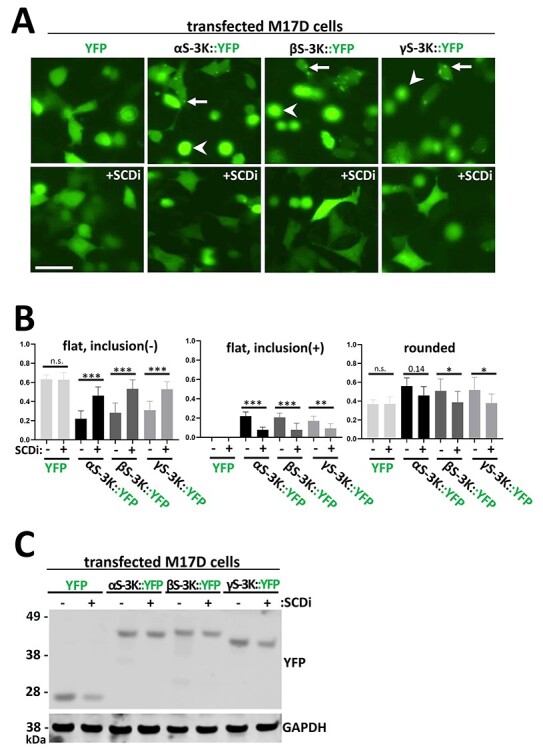
Pretreating M17D cells with SCD inhibitor rescues cellular inclusion formation and toxicity. (**A**) IncuCyte live-cell images of M17D cells transfected with either YFP alone, YFP-tagged αS-3K, βS-3K or γS-3K after 48 h. Another set of M17D transfectants was pretreated with SCD inhibitor (SCDi) MF-438 (10 μM) and was also imaged. Arrows point at inclusions, arrowheads point at rounded cells. Scale bar, 20 µm. (**B**) Quantification of the number of cells without inclusions (left graph), with inclusions (middle graph) and rounded (right graph) 48 h post-transfection and with/without 24 h pretreatment of SCD inhibitor (*y*-axis: fraction). Graph shows mean data for *N* = 3 independent experiments (*n* = 8 each) and SEM. One-way ANOVA analysis (comparisons as indicated), Tukey’s multiple comparisons test. ^*^P < 0.05; ^*^^*^P < 0.01; ^*^^*^^*^P < 0.001; n.s., non-significant. (**C**) WB of M17D cells transfected with either YFP alone, YFP-tagged αS-3K, βS-3K or γS-3K with/without pretreatment of SCD inhibitor. WB represents three independent experiments.

We recently identified by lipidomic profiling that αS cytotoxicity is accompanied by elevated levels of monounsaturated fatty acids and oleic acid (OA) in particular (41). Moreover, we also identified lipid saturation as a modifier of αS inclusion formation in a small-molecule screen (42). This prompted us to speculate about a bi-directional scenario in which αS leads to elevated OA levels, which—after incorporation into membrane lipids—increase αS membrane association and toxicity (43). Indeed, we and others have demonstrated that loss/inhibition of the OA-generating enzyme stearoyl-CoA desaturase (SCD) rescues αS-related toxicity in yeast (41,44), *Caenorhaditis elegans* (41,45), primary rodent neurons (41) and neuroblastoma cells (42). The increased cellular viability was found to be accompanied by the following changes in αS homeostasis: (1) αS serine-129 phosphorylation of αS E46K and excess wt αS was reduced upon SCD inhibition (41,42); (2) αS E46K solubility, as assessed by cytosol:membrane ratio in sequential extraction experiments, was increased (41,42) and (3) inclusion formation of αS 3K (amplified E46K) was reduced (41,42). We therefore tested if putatively beneficial effects of SCD inhibition would also hold true for the other homologs. Specifically, we focused on inclusion formation of the respective 3K variants because their intermediate effects in our experiments (Figs 3–6) indicated to us the potential for reversibility. YFP alone as well as YFP-tagged αS 3K, βS 3K and γS 3K were expressed in control versus 24 h SCD inhibitor-pretreated M17D cells (10 μM MF-438; Fig. 8A). We observed a phenotypic rescue of both inclusion-bearing (αS, βS and γS) and rounded (βS and γS) cells, as confirmed by blinded statistical analysis (Fig. 8B). While SCD inhibition did not affect the appearance of YFP-only expressing cells the percentage of flat, inclusion-free cells was markedly increased by the SCD inhibitor for all 3K transfectants. Conversely, the count of abnormal (i.e. inclusion-positive and rounded) cells was reduced (Fig. 8B), while all 3K transfectants displayed similar levels of transgene expression (Fig. 8C).

## Discussion

αS is considered a toxic protein. Its homologs βS and γS are not. In the present study, we asked if αS is truly unique among the synucleins with regard to the ability to cause cellular stress/toxicity. We found that three defined amino acid substitutions (E35K + E46K + E61K = ‘3K’) are sufficient to render all synucleins significantly more toxic than their wt counterparts. In addition to 3K, which changes three αS repeat motifs from KTKEGV to KTKKGV, we saw similar effects for motif variants KLKEGV (‘KLK’; six substitutions) as well as KLKEIV (‘EIV’; seven substitutions). All three variants have in common the expected effect of facilitating αS amphipathic helix formation at membranes (Fig. 2), thereby increasing αS membrane interactions (Fig. 3). Indeed, upon sequential extraction, we found significantly higher amounts of 3K, KLK and EIV relative to wt in detergent-soluble fractions for all synucleins, which is consistent with enhanced membrane binding (Fig. 4). The increase in membrane interaction was paralleled by reduced multimer formation, as evidenced by intact-cell crosslinking (Fig. 5). Moreover, 3K, KLK and EIV expression in neural cells triggered synuclein toxicity (Fig. 6) and inclusion formation (Fig. 7) for all three homologs.

A key motif that has been associated with αS aggregation are amino acids 61–95 (NAC domain), particularly amino acids 71–82 (20,21). The amino acid stretch 71–82 (VTGVTAVAQKTV in αS) is largely deleted in βS, and more hydrophilic in γS (VSSVNTVAEKTV). Thus, our new data are consistent with a membrane-associated mechanism of synuclein toxicity that is independent of the NAC domain and at least initially not mediated by proteinaceous β-sheet-rich protein aggregation. Related to this notion, a study by Volles *et al*. (46) compared αS *in vitro* fibrillization and yeast toxicity by screening a library of random point mutants. A lack of correlation between the two aspects suggested that fibrillization is not necessary for synuclein-induced toxicity in yeast. A second yeast screen in the same study identified 25 non-toxic αS sequence variants, which reduced membrane binding, and a toxic point mutation, which increased membrane binding (46). The authors hypothesized that yeast toxicity is caused by αS binding directly to membranes at levels sufficient to non-specifically disrupt homeostasis. A recent study performed deep-mutational αS scanning in the yeast model and likewise came to the conclusion that membrane binding mediates αS toxicity (47)**.** Previous studies had characterized the membrane-associated toxicity of αS in detail: wt αS expression in yeast causes vesicle clustering/aggregation (48) and vesicle trafficking defects (49) in the absence of apparent amyloid formation. The relevance of these findings for PD pathogenesis was highlighted when similar trafficking defects were observed in patient-derived αS A53T and triplication iPS cell cultures (50). In contrast to the yeast system, no acute toxicity was observed in the iPSC neurons, possibly owing to a better ability of mammalian cells to compensate αS-induced cellular dyshomeostasis. Interestingly, a recent characterization of Lewy body pathology came to the conclusion that excess αS interaction with lipids/membranes also plays a major role in these hallmark lesions of synucleinopathy (51).

The hypothesis that our observed synuclein toxicity is mediated by an excess of synuclein membrane interaction is further supported by our SCD inhibitor experiments (Fig. 8). SCD inhibition increases the proportion of saturated fatty acids in the membrane, while the proportion of mono-unsaturated fatty acids decreases. Higher levels of saturated fatty acids decrease membrane fluidity and thus αS membrane interaction via the formation of amphipathic helices (52). Consequently, we detected reduced inclusion formation of the membrane-affine 3K variants upon treatment with SCD inhibitor MF-438 (Fig. 8). SCD inhibition has emerged as a potential new treatment for synucleinopathies in several studies (41,42,44). The present work suggests that SCD inhibition could also overcome βS- and γS-related cytotoxicity. Yet, a disease mechanism based on βS or γS excess or misfolding that would be independent of αS has not been proposed. Our study, however, highlights that increasing βS or γS membrane binding could theoretically render these proteins toxic as well. Changing one KTKEGV motif to KLKEGV or KTKEIV might be sufficient to strengthen membrane interaction in such a way that toxicity occurs within the lifetime of a human, and it will be interesting to see if such a mutation will ever be found. In fact, two potentially DLB-linked mutations to βS (V70M and P123H) were recently suggested to have higher membrane binding affinity compared with wt βS (53). However, instead of causing neurodegeneration via βS aggregation, an indirect effect on αS aggregation had been proposed for these two mutants (8). This raises important questions about the interactions among the synuclein homologs in the cell. αS–βS interactions have been observed, most notably the aggregation-reducing effect that wt βS has over wt αS (54–57) and fPD αS mutants (58). However, there are no explicit reports on mixed helical multimers consisting of both αS and βS (or γS), and more work will be needed to establish or rule out their existence.

In our present study, we also included the engineered αS variant ‘EGR’. Based on the helical-wheel model of αS membrane interaction, this variant is expected to enhance αS cytosol localization (Figs 2 and 3). Indeed, we observed an accumulation in the cytosol upon sequential extraction (Fig. 4). EGR also reduces multimer:monomer ratios relative to wt αS, which is consistent with a model in which a specific level of αS membrane interaction is needed to keep cellular αS equilibria at bay (31). Of note, it has been suggested that native αS multimerization is a cellular mechanism of preventing αS aggregation (28). Transient αS membrane interaction seems to be critical for native αS assembly (37). Human genetics tells us that both a lack (A30P and G51D) and an increase (E46K) in αS membrane interaction are associated with PD/DLB (59). Interestingly, we observed significant toxicity for αS and γS EGR but not for βS EGR upon expression in neural cells (Fig. 6). It is tempting to speculate that the EGR-like, cytosolic αS toxicity may be mediated by ‘classical’ proteinaceous aggregation, which is facilitated by the presence of the hydrophobic αS NAC domain that is less hydrophobic in γS and partially deleted in βS (Fig. 2). However, we did not observe signs of obvious YFP-tagged or FLAG_3_-tagged αS EGR aggregation in neuroblastoma cells or rodent neurons: Figure 6 does not show focal αS accumulation, which is consistent with no or only small aggregates. Intact cell crosslinking of EGR suggests an accumulation of 14-kDa αS monomers, but it may be worth following up on an apparent increase in αS dimer formation (Fig. 5). Lastly, the analysis of EGR is further complicated by the fact that this variant apparently enhances αS expression levels (Fig. 5), precluding a firm conclusion about its toxicity relative to wt synuclein. In contrast, the αS, βS and γS 3K variants increase αS toxicity (Fig. 6) despite lower expression (Fig. 5), underlining the rapid toxicity caused by synuclein accumulation at (vesicle) membranes and/or lipid droplets.

## Materials and Methods

### cDNA cloning

FLAG_3_-tagged (3K, KLK, EIV and EGR) and untagged (wt and 3K) αS, βS and γS mutant variant constructs were designed using GeneArt Strings DNA Fragments (Life Technologies, Carlsbad, CA) and ligated into pcDNA4/TO/myc-His plasmids using the In-Fusion HD cloning system (Takara, Mountain View, CA) according to manufacturer instructions. YFP-tagged βS and γS variants were cloned using specific primers as described for αS (30).

### Primary mouse and rat neuron harvest

Primary neurons were acquired under protocol number 05022, which was approved by the appropriate Institutional Animal Care and Use Committee, the Harvard Medical Area Standing Committee on Animals. Embryos from anesthetized pregnant CD-1 mice and Sprague-Dawley rats (Charles-River Laboratories, Wilmington, MA) at embryonic day 18 were harvested by cesarean microdissection. Dissected cortices were collected in Hanks' Balanced Salt Solution on ice and dissociated with Accumax (Innovative Cell Technologies, San Diego, CA) and DNase (40 U/μl) at 37°C for 25 min followed by gentle trituration with a Pasteur pipette in Neurobasal Medium (Thermo Fisher, Waltham, MA) supplemented with B-27 (Thermo Fisher, Waltham, MA), glutamine (0.5 mM), β–mercaptoethanol (25 μM), penicillin (100 IU/ml) and streptomycin (100 μg/ml). Cells were then filtered through a 70 μM cell strainer to remove tissue debris and clumps and were seeded in flat-bottom poly-D-lysine-coated polystyrene plates (Nunc Lab-Tek 70 378–81; Thermo Fisher, Waltham, MA) at a density of 40 000 cells (per well of 96 well plate) and 200 000 cells (per well of 24 well plate). The cells were cultured by keeping half the volume of existing supplemented Neurobasal Medium in the wells and by adding a fresh half of supplemented Neurobasal Medium.

### Cell culture and transfection

Human BE(2)-M17 neuroblastoma cells (called M17D, ATCC number CRL-2267) and primary CD-1 mouse and Sprague-Dawley rat neurons (Charles River) were cultured at 37°C in 5% CO_2_. M17D cells were cultured in Dulbecco’s modified Eagle’s medium (DMEM; Thermo Fisher) supplemented with 10% FBS, penicillin (50 units per ml), streptomycin (50 μg per ml) and 2 mM L-glutamine. Cells were transfected with Lipofectamine 2000 or 3000 (Thermo Fisher) following the manufacturer’s instructions in Opti-MEM (Thermo Fisher) for M17D cells and in unsupplemented Neurobasal Medium (Thermo Fisher) for primary cells.

### Intact-cell crosslinking

Transfected cells were collected by trituration, washed with PBS and then resuspended in PBS with EDTA-free Complete Protease Inhibitor (Roche, Basel, Switzerland). Next, cells were crosslinked in 1 mM final concentration of DSG (Thermo Fisher) in DMSO for 30 min at 37°C while rotating (60). The reaction was then quenched by adding 50 mM Tris, pH 7.6, and by incubating for 15 min at room temperature (RT). Afterward, cytosolic and membrane proteins were extracted by lysing the cells PBS/protease inhibitors/1% Triton-X 100 detergent (Sigma, St. Louis, MO) by briefly vortexing followed by incubation on ice for 20 min (alternatively, sequential protein extraction was performed). Then, the lysed samples were centrifuged at 100 000×g for 1 h at 4°C to collect the supernatant.

### Immunoblotting

Protein concentrations were determined by BCA assay (Thermo Fisher), and then 20 μg of each protein sample were boiled in 1X NuPAGE lithium dodecyl sulfate (LDS) sample (Thermo Fisher) for 10 min. The samples were then run on a NuPAGE 4–12% Bis-Tris gel (Thermo Fisher). SeeBlue Plus2 marker (Thermo Fisher) was used as a protein ladder. Gels were electroblotted onto an Immobilon-Psq 0.2 μm PVDF membrane (Millipore, Burlington, MA) for 90 min at 400 mA at 4°C in transfer buffer (25 mM Tris, 190 mM glycines and 20% methanol). Then, the proteins were fixed onto the membranes with 0.4% paraformaldehyde (PFA) in PBS for 30 min at RT, rinsed with PBS and blocked in PBS/0.2% Tropix I-Block solution (Thermo Fisher) for either 30 min at RT or overnight at 4°C. Afterward, the membranes were incubated in primary antibody (in PBS/0.2% I-Block with 0.02% sodium azide) for either 1 h at RT or overnight at 4°C. Membranes were then washed three times for 30 min in PBS (containing 0.1% Tween) at RT. Then, the membranes were incubated in horseradish peroxidase-conjugated secondary antibody (GE Healthcare, Chicago, IL; diluted 1:10 000 in PBS/0.2% I-Block) for 45 min at RT. Membranes were then washed three times for 30 min in PBS/0.1% Tween and developed with SuperSignal West Dura (Thermo Fisher).

### Sequential protein extraction

Cells were first lysed by hypotonic shock (addition of H_2_O containing protease inhibitors to cell pellets, 10 min of incubation at RT while shaking). Then 10× PBS was added for a final concentration of 1×. Lysates were spun at 4°C (>12 000 g). The supernatant was collected (PBS fraction, cytosol). The pellet was lysed in PBS/1% Triton-X 100 by sonication, followed by centrifugation at 4°C (>12 000 g). The supernatant was collected (TX-100 fraction, membranes).

### Immunofluorescence

Primary rodent neurons transfected for 48 h were washed twice with Hanks' Balanced Salt Solution (Thermo Fisher) and were fixed with a PBS solution containing 4% PFA and 0.02% glutaraldehyde for 25 min at RT. The cells were then blocked and permeabilized with 5% BSA/0.25% Triton X-100/PBS for 1 h. Cells were then incubated with primary antibody (diluted in 5% BSA.PBS) for 2 h at RT or overnight at 4°C. Afterward, the cells were washed three times with PBS followed by incubating the cell with either Alexa Fluor 488- or Alexa Fluor 568-coupled secondary antibodies (Thermo Fisher; diluted 1:2000 in 5% BSA/PBS) for 1–2 h at RT. Finally, cells were washed three times in PBS for 10 min each at RT before visualizing fluorescence.

### Fluorescence microscopy

Visualizing cells by fluorescence microscopy was performed on an AxioVert 200 microscope (AxioCam MRm camera; AxioVision Release 4.8.2; all by Zeiss, Jena, Germany). Images of YFP fluorescence were collected using a GFP/FITC filter cube and were pseudo-colored green. Confocal images were obtained on a Zeiss LSM710 system.

### Antibodies

Antibodies used were monoclonal antibodies (mAbs) M2 to the FLAG tag (Sigma, 1:10 000 in WB, 1:1000 in ICC) 71.1 to GAPDH (Sigma; 1:5000 in WB) as well as polyclonal antibodies (pAbs) ab22595 to Calnexin (abcam, Waltham, MA; 1:200 in ICC, 1:1000 in WB), ab84036 to TfR (abcam; 1:1000 in WB) and anti-DJ-1 (61) (1:3000 in WB).

### Cell toxicity assay based on density

M17D cells were plated on 96 well plates and 24 h later treated with 0.1% DMSO or 10 μM SCD inhibitor (MF-438, Millipore Sigma, Burlington, MA; see Supplementary Material, Fig. S2 for structure). After this pretreatment, the cells were transfected and cell confluency was measured using the IncuCyte Zoom 2000 platform (Essen Biosciences, Ann Arbor, MI) and an IncuCyte processing definition as described before (SI Appendix, Table S2 in (42)). Quantification was performed using GraphPad Prism Version 7.

### Neuron integrity assessment

YFP-tagged wt, 3K, KLK or EGR variants for all human synuclein homologs were transfected into DIV14 rat cortical neurons via lipofection (Lipofectamine 2000 following the manufacturer’s protocol, except that unsupplemented Neurobasal media was used instead of Opti-MEM) and cells were monitored in the IncuCyte Zoom 2000 platform (Essen Biosciences). Transfection efficiency <5% allowed for assessing integrity of single transfected neurons 96 h post-transfection. After image acquisition and blinding, cells were categorized into ‘intact’ and ‘disintegrated’ and the relative percentage of intact neurons was calculated.

### Statistical analyses

Blinded analyses were performed by assigning random numbers to dishes or images by one investigator before representative images were taken or features were counted by another investigator. We performed one-way ANOVA including Tukey’s post-hoc test or unpaired two-tailed *t*-tests using GraphPad Prism Version 7 following the program’s guidelines. Normal distribution and similar variance were observed for all values. Criteria for significance, routinely determined relative to wt αS: ^*^P < 0.05, ^*^^*^P < 0.01, ^*^^*^^*^P < 0.001, ^*^^*^^*^^*^P < 0.0001. Sufficient experiments and replicates were analyzed to achieve statistical significance and these judgments were based on earlier, similar work.

## Supplementary Material

Fig_S1_2_ddab188
