# Supplementary material for: Excess membrane binding of monomeric alpha-, beta- and gamma-synuclein is invariably associated with inclusion formation and toxicity
Source: Hum Mol Genet. 2021 Jul 12;30(23):2332–46. doi: 10.1093/hmg/ddab188 (PMC8600006; doi:10.1093/hmg/ddab188)
Supplement: Fig_S1_2_ddab188 [file fig_s1_2_ddab188.pdf]

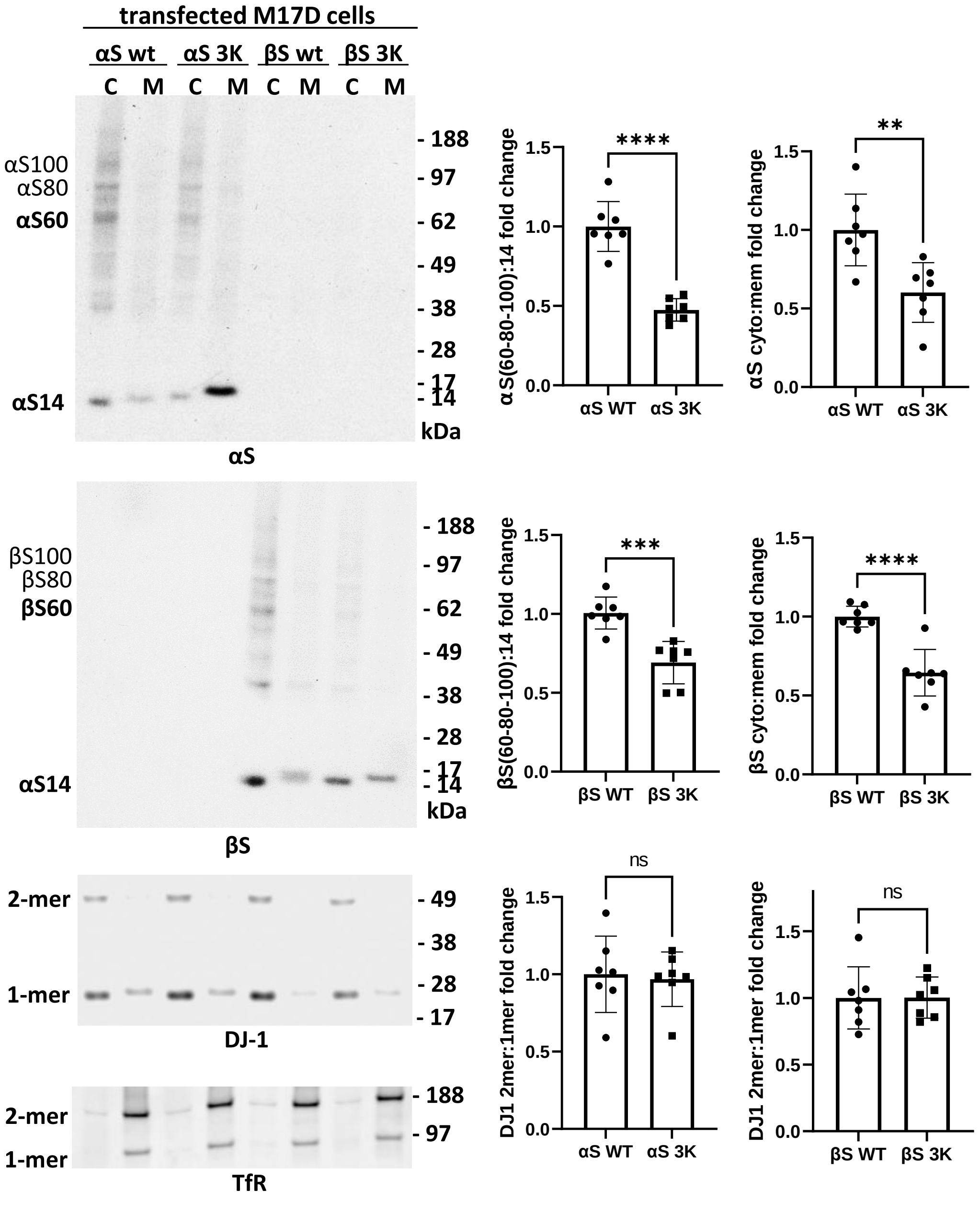

Fig. S1: αS and βS WT vs. 3K crosslinking/sequential extraction. M17D neuroblastoma cells were transfected with the indicated constructs for 48 h and subjected to 1 mM DSG crosslinking followed by sequential extraction (PBS, cytosol = C; TX-100, membrane = M). WB for αS, βS, DJ-1 and transferrin receptor (TfR). N = 2 independent experiments performed in 3 and 4 biological replicates, respectively (n = 3, 4). Graphs are means ± stdev; ****, $p < 0.0001$; ***, $p < 0.001$ **, $p < 0.01$.

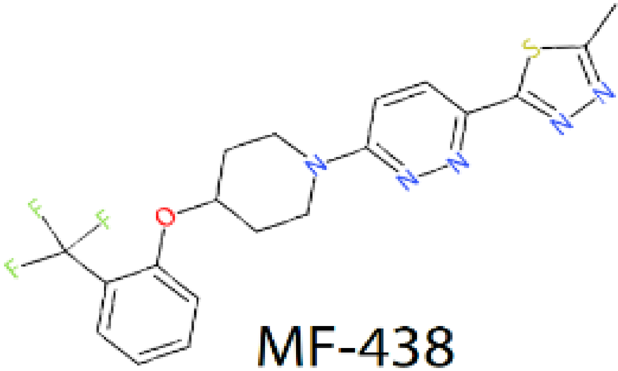

MF-438
(Millipore Sigma, 569406)

Fig. S2: Chemical Structure of the SCD inhibitor MF-438
